# The Living Age—A New Year and a New Volume

**Published:** 1898-02

**Authors:** 


### The Living Age—A New Year and a New Volume.

---

The number of *The Living Age* for the week ending January 1st, begins a new volume and a new year for that standard periodical. In this volume the striking serial story, "With all Her Heart," translated expressly for *The Living Age* from the French of Rene Bazin, will be continued until its completion. Its conclusion will be immediately followed by the publication of a new serial of unusual interest.

In 1898 it enters, with renewed interest and vitality, its fifty-fifth year. During the year now closing it has widened its scope and enlarged its volumes, enriching its pages with choice selections from the works of the most able authors and men of affairs of Continental Europe, on subjects of intelligent and timely interest, and given in a Monthly Supplement extracts from leading American magazines and readings from new books, thus establishing its claim as the reflection of the whole world's living literature.

Early numbers of the magazine will contain, among other thoughtful and important papers, one on Modern Education, by J. P. Mahaffy; M. Ferdinand Brunetiere's Impressions of America, translated for *The Living Age* from the *Revue des Deux Mondes;* some Reminiscences of Thomas Henry Huxley, by Prof. St. George Mivart; the Dual and the Triple Alliance and Great Britain, by Francis de Pressense; and the Evolution of the Idea of God, by Andrew Lang; and from week to week will appear articles by the world's foremost thinkers, investigators and authors.

The beginning of a volume is an excellent time for the beginning of a subscription, and the publishers still present to new subscribers for 1898 the eight numbers of 1897 containing the first installment of "With all Her Heart."

To every one who would keep abreast of the best thought of the time, the periodical is in fact almost a necessity. With fifty-two numbers, aggregating three thousand five hundred large pages, the subscription price, $6, is low; while for $9 the publishers offer to send any one of the $4 American monthlies or weeklies with *The Living Age* for a year postpaid.

The Living Age Co., Boston, are the publishers.

---

**Cuba, Hawaii and China** furnish the principal topics discussed editorially in the *American Monthly Review of Reviews* for February. There are also a few paragraphs of pointed comment on current domestic politics—the factional differences among Ohio Republicans and the swelling tide of Crokerism in the Democratic party. The editor gives his views on Tammany's attitude toward the New York rapid-transit problem, and on the reckless expenditure of canal-improvement funds by the Republican bosses of the State.

The editor's monthly *resume* of "The Progress of the World" opens with a presentation of the Cuban situation at the present moment. "Autonomy" is exposed as a farcical makeshift, which deceives nobody, and only serves to irritate both parties in Cuba—the insurgents and the Spanish Conservatives.

